# Interval colorectal cancer: Lesson from looking back

**DOI:** 10.1055/a-2638-6322

**Published:** 2025-07-23

**Authors:** Giovanni Aldinio, Helmut Neumann, Luigi Boni, Emanuele Dabizzi, Luca Elli, Marco Maggioni, Gian Eugenio Tontini

**Affiliations:** 1 9304 Department of Pathophysiology and Organ Transplantation, Università degli Studi di Milano, Milan, Italy; 2 Department of Interdisciplinary Endoscopy, University Hospital Mainz, Mainz, Germany; 3 9339 Department of General and Minimally-Invasive Surgery, Fondazione IRCCS Ca' Granda Ospedale Maggiore Policlinico, Milan, Italy; 4 9339 Gastroenterology and Endoscopy Unit, Fondazione IRCCS Ca' Granda Ospedale Maggiore Policlinico, Milan, Italy; 5 Department of Pathology, Foundation IRCCS Ca’ Granda Ospedale Maggiore Policlinico, Milan, Italy

Interval colorectal cancer (CRC), defined as cancer diagnosed after a negative colonoscopy but before the next recommended screening, accounts for approximately 2.8% to 4.9% of all CRCs and is predominantly located in the right colon 1 . Despite advancements in endoscopic techniques, its prevalence underscores the need for improved detection methods to reduce missed lesions.

Several strategies have been proposed to enhance adenoma detection rate (ADR), including techniques and devices for enhanced mucosal exposure, advanced imaging technologies 2 , water-aided colonoscopy, and computer-aided detection 3 4 . Retroflexion in the right colon is another technique that has gained attention as a method to improve visualization, particularly for lesions located behind folds or in difficult-to-reach areas. Numerous studies have shown that retroflexion can increase right-sided colonic ADR by 6% 5 . However, the procedure carries certain risks, including mucosal injury or perforation (0.03%) 5 , and requires relative operator expertise.

**Fig. 1:**
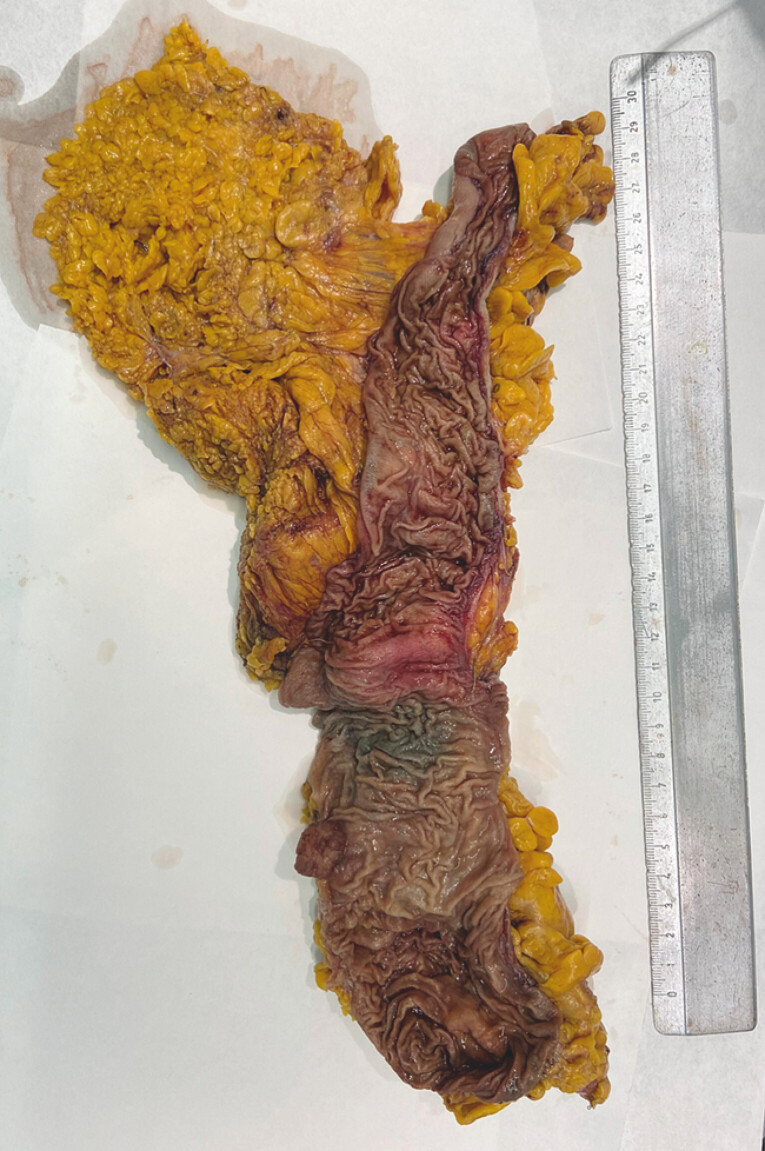
Macroscopic presentation of the right hemicolectomy surgical specimen.

**Fig. 2:**
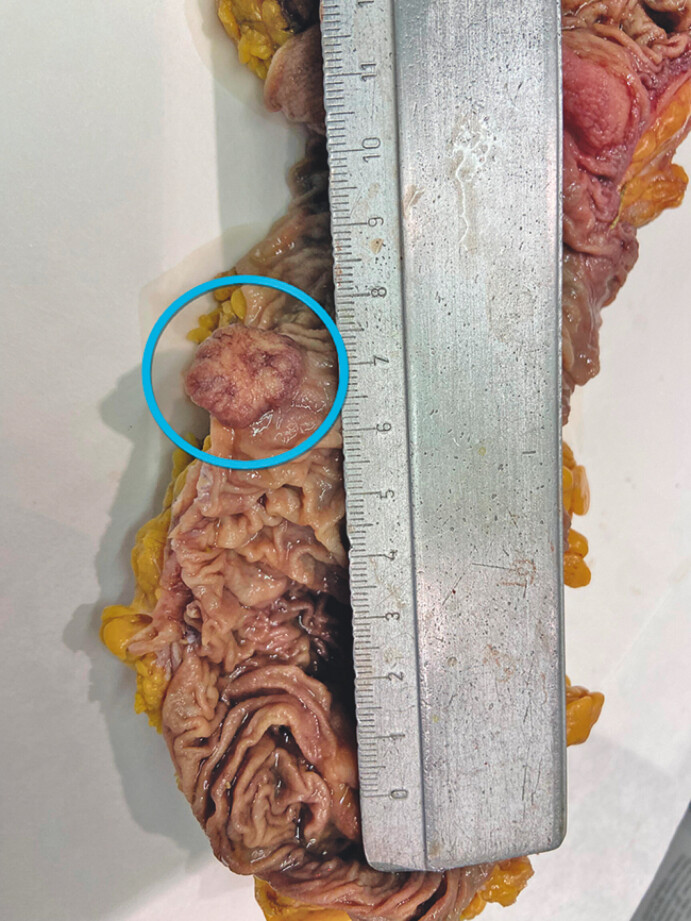
Close-up of the cancerous lesion (in the circle) in the ascending colon, located on the antimesocolic side.

**Fig. 3:**
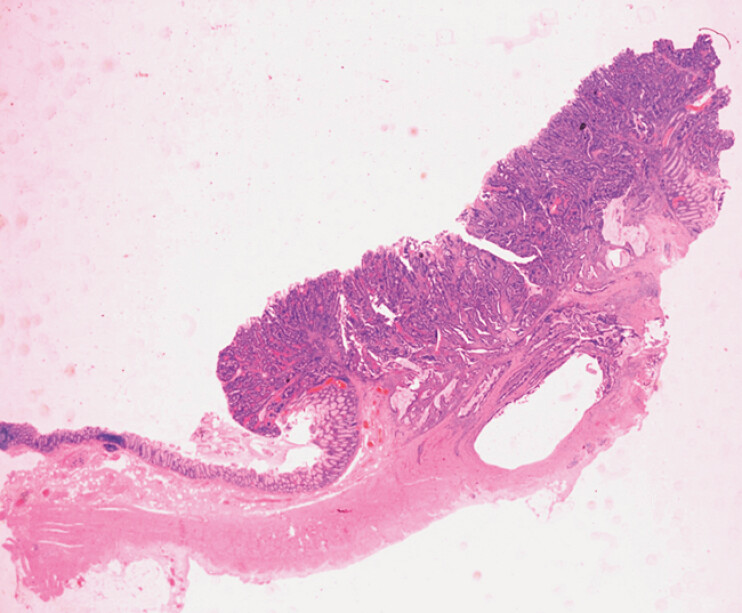
Histological overview of large bowel wall including the adenocarcinoma; hematoxylin and eosin (H&E) stain at 5x magnification.

**Fig. 4:**
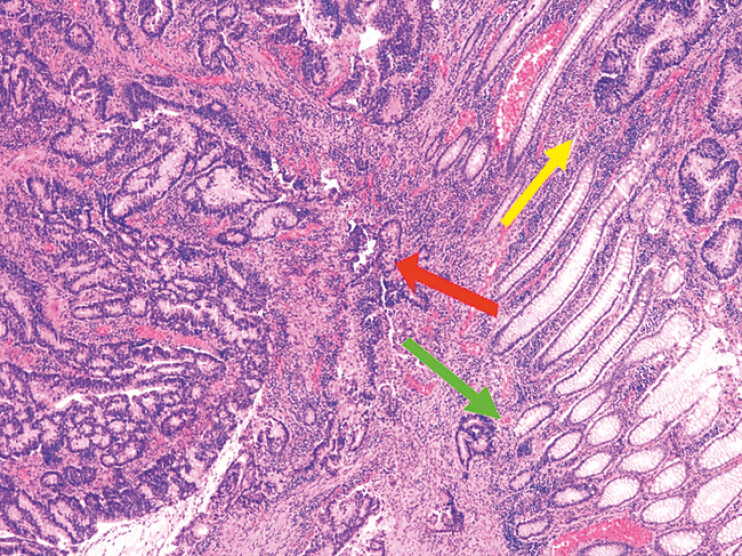
Histological border of the cancerous lesion including normal crypts in the lower right (green arrow), dysplasia at the top right (yellow arrow), and invasive adenocarcinoma on the left (red arrow); H&E stain at 40x magnification.

**Fig. 5:**
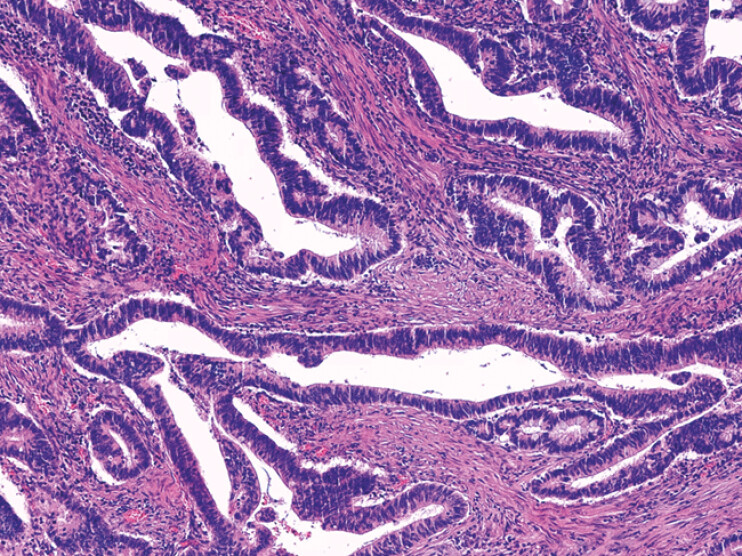
Histology of the adenocarcinoma; H&E stain at 200x magnification.

We report the case of an 83-year-old woman on a direct oral anticoagulant (DOAC) for atrial fibrillation who presented with anemia and a positive fecal occult blood test. High-definition colonoscopy 3 (Pentax Ec38-i10L, EPK-i7010) with a second and a third forward look of the right colon identified no lesion. Right colon retroflexion was performed, revealing a 20 × 15-mm non-pedunculated lesion (Paris 0-IIa) with advanced adenomatous features (Kudo V _N_ ) ( Video 1 ). Endoscopic biopsies confirmed presence of adenocarcinoma and the patient underwent right hemicolectomy (TNM: pT2, G1, N0) ( Fig. 1 , Fig. 2 , Fig. 3 , Fig. 4 , Fig. 5 ).

This video-documented case shows how retroflexion in the right colon revealed a missed colorectal cancer, highlighting its potential role in improving lesion detection.Video 1

This case provides video-documentation of a CRC diagnosis achieved through retroflexion, hereby highlighting its potential role, especially in patients with a high probability of advanced neoplasia 1 . Second, the anatomical documentation makes clear the antimesocolic region of the hepatic flexure as a challenging location for endoscopic inspection, despite standard endoscopic maneuvers to enhance blind spots. Finally, the report adds to the growing literature on endoscopic techniques to improve ADR and minimize risk of missed advanced colorectal lesions: retroflexion could be performed by operators who feel confident in its execution, with adequate instruments and in anatomical conditions that provide sufficient space for safe maneuvering.
